# Awareness and acceptability of HIV pre-exposure prophylaxis (PrEP) among students at two historically Black universities (HBCU): a cross-sectional survey

**DOI:** 10.1186/s12889-021-10996-2

**Published:** 2021-05-19

**Authors:** Nwora Lance Okeke, Tony McLaurin, Ruth Gilliam-Phillips, David H. Wagner, Valerie J. Barnwell, Yolanda M. Johnson, Osaffo James, Padonda B. Webb, Sharon D. Parker, Bendu Hill, Mehri S. McKellar, John T. Mitchell

**Affiliations:** 1 Department of Medicine, Division of Infectious Diseases, Duke University School of Medicine, Durham, NC USA; 2 Department of Psychiatry and Behavioral Sciences, Duke University School of Medicine, Durham, NC USA; 3 Department of Student Health, North Carolina Central University, Durham, NC USA; 4 Student Health Center, North Carolina A&T State University, Greensboro, NC USA; 5 Department of Social Work and Sociology, North Carolina A&T State University, Greensboro, NC USA

**Keywords:** HIV, Pre-exposure prophylaxis, PrEP, Historically black colleges and universities, Sexual health

## Abstract

**Background:**

Despite young African American adults (ages 18–24) being among the highest risk groups for HIV infection, little is known about their awareness of HIV pre-exposure prophylaxis (PrEP) – a once daily pill shown to be > 90% effective in preventing HIV. To explore awareness and acceptability of PrEP among college students in this demographic, we conducted a survey of attendees at two large historically Black universities (HBCU) in North Carolina.

**Methods:**

We administered a 14-item questionnaire to students at two HBCUs in North Carolina between February and April 2018. Questions were formatted in a yes/no or multiple choice format. Questionnaire items specifically addressed PrEP awareness and acceptability. Surveys were administered to students at a campus health fair and while transiting the campus student union via iPad. Response to all questions was optional. We fit a logistic regression model to determine association of key demographic determinants with PrEP acceptability and awareness. Statistical analyses were conducted using SAS 9.4 (SAS, Cary, NC).

**Results:**

Overall, 210 students participated in the survey, of which 60 completed all survey items as presented. The survey cohort was 75% female, 89% heterosexual and 39% freshmen. The mean age of respondents was 19.8 years (SD: 1.8). Fifty-two percent of survey respondents reported that they were aware of PrEP prior to the time of survey administration. Only 3% of respondents reported that they were on PrEP. The most common sources of information on PrEP were campus health services (24%) and non-social media advertising (15%). Of respondents who were aware of PrEP, 61% reported that they had heard about in the 6 months prior to survey administration, while only 19% say they were aware of it for more than a year. Regarding acceptability of PrEP, 58% of respondents reported that they would take a once a day pill for HIV if they were at risk. Our logistic regression analysis found no statistically significant associations between key demographic factors and PrEP awareness. However, persons who perceived themselves to be at risk for HIV acquisition were more likely to find once daily oral PrEP (relative risk 2.66 (95% CI 1.31–5.42)) as an acceptable prevention strategy than the rest of the survey cohort.

**Conclusions:**

African American HBCU students are becoming aware of PrEP, and generally perceive the intervention as acceptable and worth consideration.

**Supplementary Information:**

The online version contains supplementary material available at 10.1186/s12889-021-10996-2.

## Background

Despite a 25% decrease in new HIV infections in the United States over the last decade, the South still accounts for more than half of all new infections nationwide [1]. African Americans make up 13% of the US population [2], but accounted for 44% of all new HIV infections in 2018. These rates highlight the suboptimal implementation of novel and comprehensive HIV prevention strategies for African Americans, particularly those living in the South. Young adults (age 18–35) remain the highest risk age group for new HIV infection, making them a prime group for targeted HIV prevention-based interventions [1, 3]. Despite the significant progress that has been made in the science of HIV prevention, the difficulty in engaging young adults in HIV prevention efforts is well documented [4–6].

The most impactful advance in the HIV prevention armamentarium over the last decade has been the advent of HIV pre-exposure prophylaxis (PrEP), in the form of a once daily emtricitabine/tenofovir disproxil fumarate (FTC/TDF) or emtricitabine/tenofovir alafenamide (FTC/TAF) combination tablet [7]. Clinical trials have shown in a number of populations (e.g., men who have sex with men, HIV-serodiscordant couples, IV drug users) that when taken as prescribed, once daily PrEP is 92–98% effective in preventing HIV acquisition [8–11]. Based on these data, FTC/TDF was approved by the US Food and Drug Administration in 2012 for the indication of HIV prevention among adults [12].

Despite initially slow uptake among at-risk individuals, uptake of PrEP has rapidly improved over the last 4 years, particularly in major urban centers with robust public health infrastructure [6, 13]. The Centers for Disease Control and Prevention (CDC) recently estimated that approximately 1.1 million Americans are eligible for PrEP [14]. Recent estimates indicate that approximately 225,000 individuals at high risk for HIV infection are currently on PrEP, approximately a 200% increase from the 2016 estimate of 77,000 [15]. Unfortunately, disparities in PrEP uptake among critical risk demographics have emerged [4, 6, 16, 17]. Despite accounting for 44% of new HIV infections in 2017, African Americans represent only 11% of persons currently on PrEP [3, 18]. Similarly, despite the South accounting for 52% of all new HIV infections, PrEP utilization in the region has significantly lagged behind other regions of the country [1, 16, 18]. These data necessitate innovative interventions to rapidly increase PrEP uptake among African Americans in the South.

Historically Black Colleges and Universities (HBCUs) have served as a pillar of education for African Americans in the South for over 150 years. In 2017, the National Center of Education Statistics (NCES) estimated that HBCUs provide higher education to over 225,000 Black students [19]. Eighty-five of the 101 HBCUs currently in operation are in the South, optimally positioning them to address disparities in uptake of HIV prevention interventions among young African Americans in the region [19]. The HBCU campus often has an active campus student health services infrastructure that serves a central role in the promotion of health and wellness to their student bodies. Student groups are often embedded in these health centers as part of the health promotion arm of their operations, adding an audience-centric dimension to health promotion efforts [20].

PrEP use among young adults (age 18–24) is known to be suboptimal. A recent report states that only about 14% of persons on PrEP are age 18–24, despite this age group accounting for 21% of all new infections [21]. HBCUs provide a setting that can reach a large concentration of African American young adults living in the South. Furthermore, PrEP services could be promoted and provided to young adults via existing student health infrastructure at HBCUs. At this time, little is known about HBCU students’ awareness and acceptability of PrEP. To our knowledge, few studies have systematically examined the how PrEP is perceived among HBCU students. The aim of this study was to assess PrEP awareness and acceptability among HBCU students, and explore associations between key demographic characteristics (age, gender identity, sexual orientation) and the outcomes of interest.

## Methods

This cross-sectional survey was conducted between February 2018 and April 2018 at two HBCUs in North Carolina. PrEP awareness and acceptability questionnaires were administered at three events over the study period: twice at a booth in the campus student union during hours of peak student traffic and once at a large health promotion event on campus. All questionnaires were administered to students via iPad in brief face-to-face encounters with study staff. Participants were offered compensation for their time in the form of small gifts of less than $5 each.

The questionnaire was constructed on an online survey platform: Research Electronic Data Capture (REDCap, Nashville, TN) and administered by study staff on one of two touchscreen iPads at each site. The questionnaire had 14 items, including questions potentially hidden with branching logic based on respondent input. As a contingency when the iPads were occupied or malfunctioned, paper questionnaires following an identical format were administered. The questionnaire was divided into four sections: demographics (age, gender, academic year, sexual orientation), HIV risk perception, PrEP awareness, and PrEP acceptability.

To assess HIV risk perceptions, participants were asked about their current risk of HIV acquisition over the 3 months prior to taking the survey, based on a 4-point scale (“Not at risk”, “A little bit at risk”, Somewhat at Risk”, “Very much at risk). For the PrEP awareness section, participants were asked if they had heard of PrEP or FTC/TDF (also known as ‘Truvada’) prior to the survey. At the time of the survey administration FTC/TDF combination tablet was the only form of PrEP available in the United States. If they responded yes, they were asked for an approximation of when they heard about PrEP and the modality of how they received information on it. For the question on “how they heard about PrEP” respondents were required to choose the single best answer about their information source. PrEP acceptability, participants were asked whether they would consider taking PrEP as a once daily pill, once monthly intramuscular injection or a once every two-month intramuscular injection with the following response options: “Yes”, “No”, “Not Sure” (Additional file 1). There was a malfunction for some of the questions hidden by branching logic thus interfering with the ability of respondents to access these questions as part of the survey.

Summary statistics were calculated based on the number of respondents for each questionnaire item. We fit logistic regression models to derive bivariate and multivariate relative risks for the association of key respondent covariates for the outcomes of PrEP awareness and PrEP acceptability (SAS 9.4, Cary, NC). The awareness outcome was based on the question “Have you heard of PrEP”, presented with the binary answer (“Yes” or “No”). The acceptability outcome was based on logistic regression models for each presented modality of PrEP (once daily oral tablet, once monthly intramuscular injection, every 2-month injection). Separate regression models were fit for each of the PrEP modalities presented, and thus treated as completely independent measures. For the primary analysis, all responses of “Not Sure” were grouped with the response group “No”, and thus considered as a negative response (“Not sure= ‘No”). All study activities were reviewed and approved by the Duke University School of Medicine Institutional Review Board (IRB) and in coordination with the IRBs of the participating HBCUs. All study activities were carried out in accordance with relevant guidelines and regulations and to the standards of the aforementioned regulatory bodies.

## Results

Overall, 210 students responded to the survey. Among participating students, 158 (75%) were women, and the mean age was 19.8 years (SD = 1.8). Regarding sexual orientation, 186 (89%) self-reported as “heterosexual”. Eighty-one of the study participants (39%) were freshman, 47 (22%) were sophomores, 51 (24%) were juniors, 23 (11%) were seniors, 5 (2%) were graduate students, and 3 (1%) declined to respond (Table 1). Unfortunately, an equipment malfunction with the survey entry apparatus led to numerous items on the survey left unanswered by a majority of respondents. This malfunction affected responses of most of the participants at one of the universities and approximately half at the second university. Given the similarities in demographics of the two sites, the authors do not believe that the gating error materially affects the interpretation of our results (*n* = 210 for survey participants; *n* = 60 for participants who completed entire survey). In comparing respondents who did not complete the entire survey (*n* = 150) to respondents who completed the entire survey (*n* = 60), the only demographic factor that reached statistical significance was the proportion of seniors in each group (18% in completers, v. 8% in non-completers, *p* = 0.03; Additional file 2). The malfunction also did not affect the answers required for the logistic regression analysis presented in this section (*n* = 210). Specifically, the malfunction omitted responses to three of the 14 survey items: “Are you on PrEP?”, “Where did you hear about PrEP?” and “How long have you known about PrEP?”

**Table 1:** Demographics of Survey Respondents (*n* = 210)

Characteristic	All (***n*** = 210) (%)
**Gender**	
**Male**	53 (25)
**Female**	158 (75)
**Transgender**	0
**Mean Age (SD)**	19.8 (1.8)
**Sexual Orientation**	
**Straight**	186 (89)
**Gay or Lesbian**	5 (2)
**Bisexual**	9 (4)
**Other**	6 (3)
**Decline to Answer**	4 (2)
**Year in College**	
**Freshman**	81 (39)
**Sophomore**	47 (22)
**Junior**	51 (24)
**Senior**	23 (11)
**Graduate Student**	5 (2)
**Decline to Answer**	3 (1)

A majority of respondents (52%) were aware of PrEP prior to the time of survey administration. Of persons who knew about PrEP, 39% reported that they first heard about PrEP in the 3 months prior to survey administration. Only 19% of respondents reported that they aware of PrEP for ≥1 year before the survey. Twenty-four percent of respondents reported that they found out about PrEP from their student health clinic, and 17% reported that they heard about it from a student-health sponsored health promotion event. Another 15% of respondents reported that they first became aware of PrEP through social media. Only two of 60 respondents who answered the question on whether or not they were on PrEP reported that they were currently on it (Table 2).

**Table 2:** Student responses to survey on PrEP awareness

Characteristic	N (%)
**Have you heard of PrEP? (*n*** **= 210)**	
**Yes**	110 (52)
**No**	100 (48)
**Are you on PrEP? (*n*** **= 60)**	
**Yes**	2 (3)
**No**	58 (97)
**Where did you hear about PrEP? (*n*** **= 54)**	
**Friend/sex partner**	4 (7)
**Health promotion event on campus**	9 (17)
**Student Organization**	2 (4)
**Advertisement (not social media)**	8 (15)
**Social Media**	4 (7)
**Student Health**	13 (24)
**In class**	2 (4)
**Can’t remember/decline to answer**	10 (19)
**Other**	2 (4)
**How long have you known about PrEP? (*n*** **= 54)**^**a**^	
**< 3 months**	21 (39)
**3–6 months**	12 (22)
**6–12 months**	11 (20)
**1–2 years**	7 (13)
**2+ years**	3 (6)
**Based on your behavior in the last 3 months, do you think that you are at risk to get HIV? (*n*** **= 210)**	
**Not at risk**	153 (73)
**A little bit of risk**	33 (16)
**Somewhat at risk**	14 (7)
**Very much at risk**	4 (2)
**Decline to Answer**	6 (3)
**Would you take a pill once a day to protect yourself from getting HIV? (*n*** **= 210)**	
**Yes**	122 (58)
**No**	39 (19)
**Not Sure**	49 (23)
**Would you take an injection once a month to protect yourself from getting HIV? (*n*** **= 210)**	
**Yes**	107 (51)
**No**	57 (27)
**Not Sure**	46 (22)
**Would you take an injection once every two months to protect yourself from getting HIV (*n*** **= 210)**	
**Yes**	120 (57)
**No**	47 (22)
**Not Sure**	43 (20)
**What method of PrEP would you prefer to use? (*n*** **= 210)**	
**Pill once a day**	62 (29)
**Injection once a month**	30 (14)
**An injection once every two months**	79 (38)
**Not sure**	39 (19)
**Expressed interest in at least one form of PrEP? (*n*** **= 210)**	
**Yes**	145 (69)
**No**	65 (31)

^a^Sample size varies for each item due to equipment malfunction

Regarding self-perceived risk for HIV acquisition, 73% of respondents felt that they were “not at all at risk” for HIV based on their current behavior. Sixteen percent of respondents deemed themselves at slight risk of HIV infection. Only 9% of respondents considered themselves “somewhat at risk” or “very much at risk”. Two percent of respondents declined to answer the question (Table 2).

A majority of respondents reported that they would consider taking PrEP as a once daily pill (58%). The same proportion of respondents reported that they would consider an injection once every 2 months to prevent HIV (57%). A smaller majority (51%) reported that they would accept a once monthly injection. When asked which of the three options they felt was most preferable, 38% reported that they would prefer an injection every 2 months, while 29% reported that their preference would be a once daily pill. Nineteen percent of respondents reported that they were not sure. Overall, 69% of surveyed students found at least one administration method of PrEP as acceptable (Table 2).

Gender, age, academic year, sexual orientation, and perceived risk were not significantly associated with PrEP awareness in the regression analysis (Table 3). In the logistic regression analysis for the acceptability outcome, persons who perceived themselves as at increased risk for HIV acquisition were more likely to perceive once daily PrEP as an acceptable prevention intervention (relative risk 2.66 (95% CI 1.31–5.42), *p* = 0.007) than the rest of the survey cohort. There also appeared to be a trend towards this group seeing a once monthly injection as an acceptable prevention intervention, but the association did not reach statistical significance (RR 1.80 (95% CI 0.93–3.44), *p* = 0.08) (Table 4).

**Table 3:** Relative risk of PrEP awareness associated with selected demographic characteristics

Characteristic	Relative Risk (95% CI)		*p*-value
	Bivariable	Multivariable	
**Male Gender**	0.87 (0.55–1.36)	0.81 (0.41–1.57)	0.53
**Age (per 1 year increasae)**	0.95 (0.85–1.07)	0.93 (0.75–1.16)	0.54
**Freshman**	1.14 (0.78–1.67)	1.01 (0.20–4.98)	0.99
**Sophomore**	1.04 (0.54–1.99)	0.94 (0.20–4.47)	0.94
**Junior**	0.68 (0.36–1.28)	0.85 (0.19–3.83)	0.84
**Senior**	0.99 (0.55–1.81)	1.04 (0.21–5.31)	0.96
**Non-Heterosexual Orientation**	1.05 (0.56–1.97)	1.10 (0.42–2.84)	0.84
**Perceived Risk**	1.05 (0.69–1.60)	1.11 (0.60–2.09)	0.74

**Table 4:** Adjusted relative risks for factors associated with acceptability of PreP by mode and frequency of administration

Characteristic	Daily PrEP RR (95% CI)	***p***-value	Monthly Injection = (RR 95% CI)	***p***-value	Every 2 month injection (RR 95% CI)	***p***-value	Any Prep RR (95% CI)	***p***-value
**Male Gender**	0.74 (0.37–1.48)	0.40	0.67 (0.34–1.33)	0.25	0.62 (0.31–1.21)	0.16	0.78 (0.38–1.60)	0.51
**Age (per 1 year increase)**	0.98 (0.79–1.23)	0.90	1.14 (0.91–1.42)	0.25	1.10 (0.88–1.37)	0.40	1.05 (0.83–1.33)	0.70
**Freshman**	1.24 (0.60–2.57)	0.55	1.72 (0.84–3.51)	0.13	1.80 (0.89–3.69)	0.10	1.33 (0.62–2.86)	0.45
**Sophomore**	1.03 (0.51–2.05)	0.92	0.68 (0.35–1.35)	0.27	0.82 (0.41–1.60)	0.56	0.92 (0.45–1.88)	0.82
**Junior**	0.83 (0.40–1.73)	0.63	0.76 (0.37–1.55)	0.45	0.79 (0.38–1.60)	0.51	0.69 (0.32–1.49)	0.35
**Senior or higher**	1.60 (0.59–4.38)	0.35	1.74 (0.65–4.66)	0.26	1.43 (0.53–3.83)	0.48	2.32 (0.70–7.67)	0.17
**Non-Heterosexual Orientation**	2.27 (0.76–6.81)	0.14	1.28 (0.48–3.37)	0.62	1.22 (0.45–3.29)	0.69	2.51 (0.69–9.11)	0.16
**Perceived Risk**	**2.66 (1.31–5.42)**	**0.007**	**1.80 (0.93–3.44)**	**0.08**	1.23 (0.64–2.38)	0.52	1.60 (0.77–3.32)	0.21

## Discussion

In our survey of 210 students at two HBCUs in the US South, approximately half of the respondents were aware of PrEP prior to survey administration. Interestingly, 61% of respondents learned about PrEP in the 6 months leading up to the survey suggesting a recency of PrEP awareness among the survey cohort. The overall perception of risk of HIV acquisition in among the sample was low (89% perceived themselves as low or no risk) especially considering that almost 70% of surveyed students reported that they would consider at least one administration method of PrEP. Our findings are consistent with other reports in the literature suggesting that few HBCU students perceive themselves at increased risk for HIV acquisition [22, 23]. One such study, a 2011 survey of 1230 HBCU students, reported that 79% of respondents perceived themselves as low risk for HIV acquisition [23]. Our results contribute to the literature, and although the fact that HIV risk perception in the interval between the cited studies remained relatively stable is somewhat surprising, it reinforces the need for continued efforts towards education on HIV prevention and overall sexual wellness in this key population.

To our knowledge, our study is the first to assess acceptability and awareness of PrEP among HBCU students. It also represents one of the first studies to inquire on the acceptability of potential methods of PrEP administration among college students. This is important since HBCUs are predominantly clustered in the Southern US where there is a disproportionately higher rate of HIV and contain a student body primarily composed of African American young adults, which is a group at higher rate for HIV as well. Although we recognize that survey data from the two institutions involved in the study cannot be generalized to all HBCU students in the South, findings from our study provide important insights into the state of PrEP awareness among this critical demographic.

In spite of the ongoing public health crisis that the “Southern HIV epidemic” presents, research on the epidemiology of HIV among students on HBCU campuses is minimal [24, 25]. Prior reports in the literature have primarily been centered around general HIV knowledge of HBCU students, risk perception and correlates of high-risk behavior [22, 23, 26–28]. One study surveyed health administrators on HBCU campuses regarding their perception of institutional HIV prevention strategies on their campus and half reported no formal campus HIV prevention policy existed [29]. Although the study was published in 2011 prior to FDA approval of FTC/TDF for PrEP, other HIV prevention strategies were available. Our findings among others highlight the need for the development of formal strategies informed by current student aptitude on HIV prevention strategies, students’ perception of HIV acquisition risk and the state of the epidemic in the regions that the institutions occupy. In the face of a persistent epidemic in the South, more studies are needed to augment the body of knowledge for HBCU and campus leadership to build comprehensive and evidence-based HIV prevention strategies best suited for HBCUs.

In our survey, half of respondents reported that they were aware of PrEP prior to the time of the survey. The level of awareness among our patient sample is high compared to similar surveys of PrEP awareness among young Black adults [4, 30, 31]. One study reported by Ojikutu et al. administered in 2016 reported that PrEP awareness among 855 Black adults surveyed (median age = 33.6 years) was 14.5%. Notably, among MSM in the same survey, awareness of PrEP was 51.6% [32]. Due to the very low self-report rate of gay, lesbian or bisexual orientation, we were unable to determine similar differences in PrEP awareness by sexual orientation grouping. The awareness among young Black women is not as well documented, making the fact that our survey sample was 75% women unique. The higher level of awareness of PrEP in this group may be due to a higher level of education, and health literacy as a result, among college students [33]. We acknowledge that this may affect the generalizability of the findings among this demographic as a whole. The timing of when our survey was administered is also important to consider, in the setting of renewed efforts towards marketing PrEP more aggressively to young adults, including college students [34]. The impact of the student health services as an effective promotor of PrEP in this particular subset of young Black adults is also consequential as suggested by our findings that 41% of respondents reported that they heard about PrEP from events associated with their campus student health services. Although the level of PrEP awareness among young Black adults (particularly young Black women) as suggested by our data is encouraging, continued efforts to develop comprehensive and sustainable HIV prevention and sexual wellness promotion strategies centered around campus student health services are critical.

Another interesting observation from our study is the low level of perceived risk of HIV acquisition among the study population. In our survey, only 27% of respondents perceived themselves at any risk at all for HIV infection. Despite accounting for 12% of all new HIV infections in the United States, the perceived risk of HIV among young Black adults remains persistently low [1, 22, 35–37]. Several reports have documented this trend over the last decade. In a recent survey of 1617 young Black teens and adults (age 14–21), only 34% of respondents perceived themselves to be at risk for HIV infection. The study found that when perceived risk was compared with historical epidemiologic risk, there was significance discordance [38]. Recent qualitative reports suggest that among Black female college students in particular perceive pregnancy as more of a threat than sexual transmitted infection (STI) acquisition [39, 40]. Findings from these reports suggest that these women perceived avoidance of sex outside a monogamous relationship as the prime risk factor for STI risk reduction, and thus were less likely to use condoms if they were in a monogamous relationship [39–41]. Campus sexual wellness programs should focus on the importance of accounting for the unobserved sexual behaviors of “monogamous” partners in assessing one’s own risk for STIs including HIV. In marketing PrEP to HBCU students, this consideration is of particular importance given the well-documented gender imbalance (predominance of female students) at most historically-Black institutions in the US, corroborated by our survey sample [42]. Although only a small subset of the total number or respondents, persons who perceived themselves at risk appeared to be more open to once daily PrEP for HIV prevention, suggesting some concordance between risk perception and acceptability of PrEP (Table 4). Further studies should explore this potential link further.

Our report presents novel data about the acceptability of injectable PrEP among young adults. Phase 3 trials of injectable long-acting cabotegravir for the indication of HIV pre-exposure prophylaxis are ongoing, but barring unanticipated failures, PrEP may be widely available as an injection every 8 weeks in the near future [43, 44]. As a result, understanding how injectable PrEP would change the acceptability of PrEP among at-risk individuals is essential. Three recent reports have shown that among at-risk MSM, injectable PrEP every 8 weeks is preferred to a daily pill [45–47]. To our knowledge, our study presents the largest survey of PrEP acceptability among young Black university students to date. Findings from our study confirmed the preference of injectable PrEP over a once daily pill (38% v. 29%, respectively). Interestingly, the daily pill was seen as more desirable than a once monthly injection (29% v. 14%, respectively), owing perhaps to an over-representation of women in our study sample, with the assumption that women are more amenable to daily prophylaxis given their experience with oral contraception. These results suggest that injectable PrEP will significantly change the landscape of PrEP acceptability among HBCU students nationwide. It is also especially encouraging that a majority of our respondents (69%) expressed some interest in any form of PrEP.

Our study has a number of limitations. The sample size was too small to clearly define differences in responses among key at-risk groups (e.g., only 14 respondents self-reported as gay, lesbian or bisexual). It also appears that sample may have been underpowered to detect a real difference in some of the outcomes, as is apparent in the PrEP acceptability results among men and persons who perceive themselves as at-risk for HIV acquisition (Table 4). Also, administering the survey in the setting of a health promotion event may bias the overall sample towards a sample of higher health literacy and awareness. However, the health literacy event that we attended was in a very “high traffic” area, and it is unlikely that there was a significant bias of attendees to the event given its location on campus. It is also important to note that while we sampled students from two large HBCUs in the South, there is no clear indication that our sample was representative of all HBCU students in the region. Furthermore, our findings cannot be generalized to all young, Black adults in the South, particularly those who are not college students, or Black adults who are students at predominantly White institutions (PWI).

## Conclusions

PrEP continues to emerge as an important part of the HIV prevention toolbox [48]. Our study suggests that students on two HBCU campuses are not only aware of PrEP but also find it acceptable and worth consideration. Campus student health services play a central role in promoting HIV risk reduction strategies and the broader message of sexual wellness overall to their respective student bodies. Our data highlights the recency of PrEP awareness among HBCU students which although promising, further emphasizes the need to build upon the emerging momentum of PrEP dissemination on HBCUs with campus stakeholder-led, comprehensive PrEP implementation strategies that directly address the unique needs of their student populations.

## Supplementary Information

**Additional file 1.** Study Survey Instrument.

**Additional file 2. **Demographics of survey sample based on survey completion status (*n* = 210).

## Data Availability

The datasets used and/or analyzed during the current study are available from the corresponding author on reasonable request.

## Abbreviations

FTC/TDF: Emtricitabine/tenofovir disproxil fumarate
HBCU: Historically Black colleges and universities
HIV: Human immunodeficiency virus
MSM: Men who have sex with men
PrEP: Pre-exposure prophylaxis
STI: Sexually transmitted infection
