# Supplementary material for: Awareness and acceptability of HIV pre-exposure prophylaxis (PrEP) among students at two historically Black universities (HBCU): a cross-sectional survey
Source: BMC Public Health. 2021 May 19;21:943. doi: 10.1186/s12889-021-10996-2 (PMC8132367; doi:10.1186/s12889-021-10996-2)
Supplement: Supplementary file 1 — Additional file 1. Study Survey Instrument. [file 12889_2021_10996_MOESM1_ESM.docx]

**Appendix A**. Study Survey Instrument

Thank you for participating in this survey. The objective of this survey is to assess how much students on your campus know about HIV prevention strategies available to them. The questionnaire will take 5-10 minutes to complete. It is completely anonymous (unless you provide your email address to be contacted by the study team). Otherwise, there will be no information linking you the responses you give in the questionnaire. Data collected in this survey will not be made available to anyone outside the student health clinic staff and the study team. f the survey indicates implied consent to participate.

Your participation is voluntary and you may choose to discontinue participation at any time. If you wish to decline, simply click out from your browser window. There will be no negative consequences for not completing the questionnaire.

There will be direct compensation for completing the survey. However, please take a picture of the unique confirmation code. The survey administrator will record the code, and you will be entered into a raffle to win a prize at the conclusion of the survey period.

Please contact Dr. Lance Okeke at lance.okeke@duke.edu or the Duke IRB at (919) 668-5111 with any questions or concerns consequences as a result of not completing the survey.

Have you completed this questionnaire before?

- Yes
- No

Sex at Birth

- Male
- Female

How would you identify your sexual orientation?

- Homosexual
- Heterosexual
- Bisexual
- Not Sure
- Decline to Answer

How old are you? (enter free text)

What class are you in?

- Freshman
- Sophomore
- Junior
- Senior
- Graduate Student
- Decline to Answer

Have you heard of the once daily pill to prevent HIV (a.k.a PrEP, Truvada)?

- Yes
- No

If Yes, where did you hear about PrEP from?

- A friend or a sex partner
- An advertisement/commercial (TV, magazine, online, billboard)
- Student Health
- A health promotion event on campus
- A student organization on campus
- Social media
- Other (write in)
- Decline to answer

If Yes, how long ago did you hear about PrEP?

- Within the last 3 months
- 3-6 months ago
- 6-12 months ago
- Over a year ago
- Over two years ago

Based on your behavior over the past 3 months, how much do you think you are at risk for getting HIV?

- Not at risk
- A little bit at risk
- Somewhat at risk
- Very much at risk

How likely do you think you are to get HIV in your lifetime?

- Not at risk
- A little bit at risk
- Somewhat at risk
- Very much at risk

Would you take a pill once a day, every day to protect yourself from getting HIV?

- Yes
- No
- Not Sure

Would you take an injection once a month to protect yourself from getting HIV?

- Yes
- No
- Not Sure

Would you take an injection once every two months to protect yourself from getting HIV?

- Yes
- No
- Not Sure

If you were to take a medication to prevent yourself from getting HIV, which method would you prefer to use?

- A pill once a day, every day
- An injection once a month
- An injection once every two months

If you are on PrEP and would like to be contacted for participation in another study on adherence to PrEP with a mobile app, please enter your e-mail address here:
