# Supplementary material for: Awareness and acceptability of HIV pre-exposure prophylaxis (PrEP) among students at two historically Black universities (HBCU): a cross-sectional survey
Source: BMC Public Health. 2021 May 19;21:943. doi: 10.1186/s12889-021-10996-2 (PMC8132367; doi:10.1186/s12889-021-10996-2)
Supplement: Supplementary file 2 — Additional file 2. Demographics of survey sample based on survey completion status (n = 210). [file 12889_2021_10996_MOESM2_ESM.docx]

**APPENDIX B.** Demographics of survey sample based on survey completion status (n = 210)

| Characteristic | All  (n = 210) (%) | Completed survey (n=60) | Did not complete survey  (n = 150) | p-value (comparison between groups) |
| --- | --- | --- | --- | --- |
| Gender |  |  |  |  |
| Male | 53 (25) | 20 (33) | 33 (22) | 0.08 |
| Female | 158 (75) | 40 (67) | 118 (79) | 0.07 |
| Transgender | 0 | 0 | 0 |  |
| Mean Age (SD) | 19.8 (1.8) | 20 (2.0) | 19.6 (1.8) |  |
| Sexual Orientation |  |  |  |  |
| Straight | 186 (89) | 51 (87) | 135 (90) | 0.30 |
| Gay or Lesbian | 5 (2) | 2 (3) | 3 (2) | 0.57 |
| Bisexual | 9 (4) | 3 (5) | 6 (4) | 0.32 |
| Other | 6 (3) | 3 (5) | 3 (2) | 0.23 |
| Decline to Answer | 4 (2) | 1 (2) | 3 (2) | 0.87 |
| Year in College |  |  |  |  |
| Freshman | 81 (39) | 23 (38) | 58 (39) | 0.96 |
| Sophomore | 47 (22) | 12 (20) | 35 (23) | 0.60 |
| Junior | 51 (24) | 11 (37) | 40 (27) | 0.20 |
| Senior | 23 (11) | 11 (18) | 12 (8) | **0.03** |
| Graduate Student | 5 (2) | 2 (3) | 3 (2) | 0.57 |
| Decline to Answer | 3 (1) | 1 (2) | 2 (1) | 0.85 |
